# Balancing Equity in General Chemistry Laboratory Courses: The Complex Impact of Specifications Grading on Student Success and Opportunity Gaps

**DOI:** 10.1021/jacsau.5c00210

**Published:** 2025-05-19

**Authors:** Brandon J. Yik, Lisa Morkowchuk, Lindsay B. Wheeler, Josipa Roksa, Haleigh Machost, Marilyne Stains

**Affiliations:** † Department of Chemistry, 2358 University of Virginia, Charlottesville, Virginia 22904, United States; ‡ Department of Chemistry, 1355 University of Georgia, Athens, Georgia 30602, United States; § Center for Teaching Excellence, University of Virginia, Charlottesville, Virginia 22904, United States; ∥ Department of Sociology and School of Education and Human Development, University of Virginia, Charlottesville, Virginia 22904, United States

**Keywords:** alternative grading, chemistry education research, testing and assessment, equity, first generation, transfer student

## Abstract

Specifications grading has been proposed as an alternative grading method to better promote student success over traditional grading schemes. Within the chemistry community, specifications grading has been growing in popularity over the past decade as demonstrated by the rise of publications and conference talks. While several studies describe shifts in the final grade distribution as a result of the implementation of specifications grading, no study explores the differential impact on students of different social identities. In this study, we analyze over 9700 final course grades of a year-long general chemistry laboratory course under both traditional and specifications grading schemes. Data are analyzed by individual student’s social identities (i.e., gender, generation status, underrepresented minority status, and transfer student status) and students’ intersectional identities. Our results are mixed and conflicting. More systemically minoritized students pass these courses with high grades under specifications grading, but opportunity gaps between systemically minoritized students and their systemically advantaged counterparts remain. The results of this implementation show that the impact of specifications grading on students is complex and that much still needs to be understood about students’ experiences with different grading schemes and their impact.

## Graphical abstract

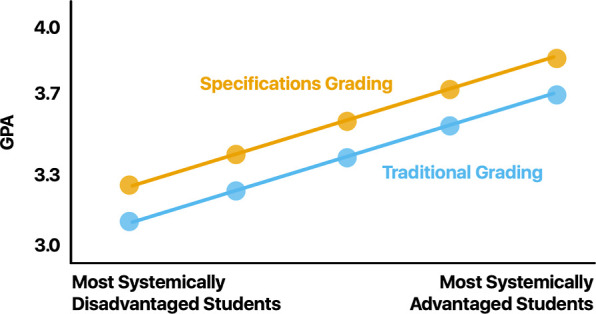

## Introduction

Prior to the 1930s, grades primarily served the purpose of internal communication of students’ achievement and knowledge. As education became more integrated across different institutions, the A–F and 100-point grading systems grew in popularity since they allowed for the comparison of students between different schools.^1,2^ Since then, grades have played a significant role in students’ education and lives. For example, grades are tied to receiving scholarships and financial aid and may thus put in jeopardy a student’s ability to pursue postsecondary education. Grades can influence students’ decisions about their major, which then affects their timeline to earn a postsecondary degree and be accepted to graduate or professional schools.^3–7^ Given the critical role that grades play in student retention, it is most unfortunate that extensive research has demonstrated the inherent flaws of the traditional grading systems including their contribution to educational inequities.^8–11^ For example, implicit racial, class, and gender biases among other systemic issues can negatively affect the grades assigned to systemically minoritized students.^12^ That is, the historical practice of selecting students for higher education has enabled systemic structures that are noninclusive of the lived experiences of minoritized students.^13,14^ A grading reform movement in science, technology, engineering, and mathematics (STEM) undergraduate education over the past decade has emerged to address the reported flaws of the traditional grading system. This movement helped raise STEM instructors’ awareness of alternative grading practices, including mastery grading, standard-based grading, and specifications grading.^15^

### Specifications Grading

Specifications grading, or specs grading, combines different aspects of alternative grading practices including mastery learning, contract grading, and competency-based education.^16^ Specifications grading emphasizes mastery of course content through well-defined expectations using a pass/fail grading approach for individual assignments or learning outcomes. Features of specifications grading include: (1) individual assessments are graded as pass/fail based on clear passing expectations that aligns with the learning outcomes for the course, (2) passing assignments reflect at least B-level work in a traditional grading system, (3) students are allowed to revise assessments that do not yet meet passing expectations for a limited number of assignments, and (4) final grades are determined through bundles of assessments with higher course grades requiring students to demonstrate a more advanced mastery of content and/or skills (Figure 1).^16^ While this is how specifications grading is formalized, instructors have modified this alternative grading scheme to fit their course contexts and needs.^17^

**Figure 1:**
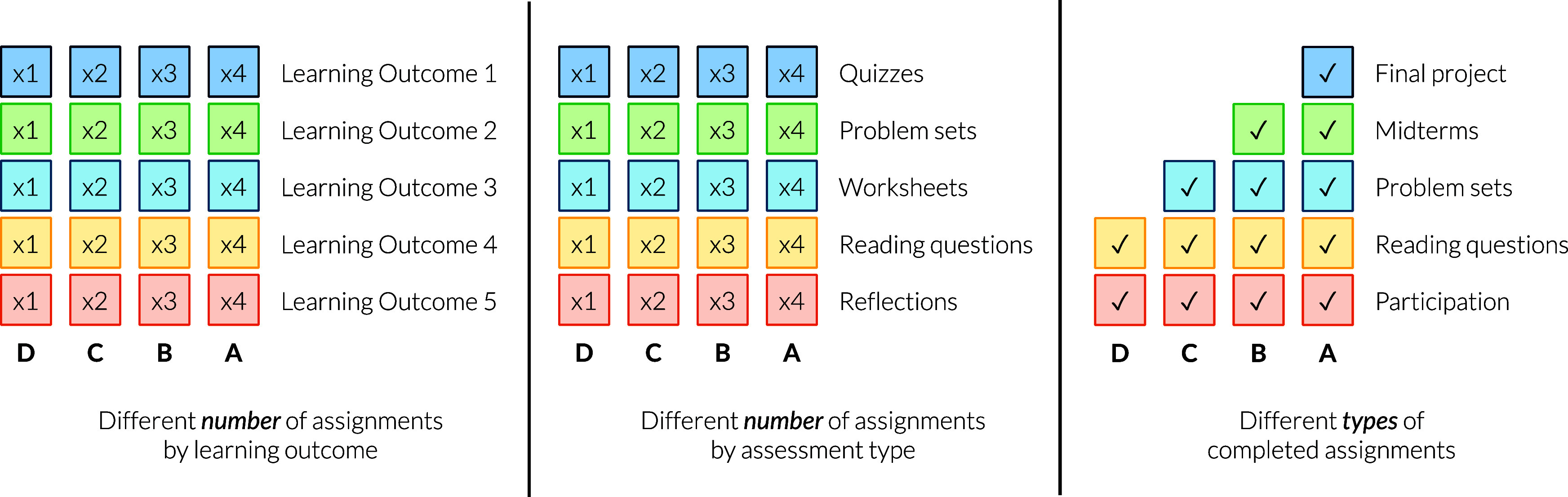
Examples of bundling strategies in specifications grading. Columns represent bundles and their associated course-level letter grade, and rows represent different learning outcomes or types of assignments. The number of times an assignment must be passed is represented by a numerical value; for example, a box with “x2” indicates that the assignment must be passed twice. A √ represents that there is only one of that assignment type, and it must be passed. Reproduced with permission from ref 18, CC-BY 4.0.

Specifications grading is the alternative grading practice that predominates in chemistry with implementations reported in nearly all chemistry subdisciplines.^19–34^ The rapid rate of adoption among postsecondary chemistry instructors since specifications grading was first formalized by Nilson in 2015 has been highlighted in a Chemical & Engineering News article,^35^ an American Chemical Society Office of Higher Education newsletter article,^36^ and is exemplified by the exponential growth in the number of presentations on the practice at the Biennial Conference on Chemical Education (from 8 during the 2017–2020 period to 58 from the 2021–2024 period). Moreover, chemistry instructors who have tried implementing specifications grading are often committed to continuing and expanding its use in other chemistry courses.^21^ Specifications grading is thus not only here to stay, but its adoption is expected to continue to rise. Therefore, it is timely and essential to understand its effectiveness and optimize its impact on student outcomes in our chemistry courses.

### Empirical Investigations of Specifications Grading

Nilson, who first formalized specifications grading in her 2015 book, claims that specifications grading achieves 15 outcomes:^16^ (1) uphold high academic standards, (2) reflect student learning outcomes, (3) motivate students to learn, (4) motivate students to excel, (5) discourage cheating, (6) reduce student stress, (7) make students feel responsible for their grades, (8) minimize conflict between faculty and students, (9) save faculty time, (10) give students feedback they will use, (11) make expectations clear, (12) foster higher-order cognitive development and creativity, (13) assess authentically, (14) have high inter-rater agreement, and (15) be simple. However, specifications grading has largely not been formally evaluated,^37–39^ and there are only a few reports in the chemistry education literature that relate to some of Nilson’s outcomes.^18,21,25,26,30^

### Empirical Investigations of Nilson’s Outcomes

The first empirical measure of some of Nilson’s outcomes was recently developed.^18^ The focus of this empirical measure, the Perceptions of Grading Schemes (PGS) instrument, was on student-related outcomes: reflect student learning outcomes, motivate students to learn, motivate students to excel, reduce student stress, give students feedback they will use, and make expectations clear. The PGS instrument was collected from students enrolled in the first and second semester of a general chemistry laboratory course (*n* = 1355). Mixed results emerged from this study: students perceived specifications grading in their course to provide a lower anxiety environment and clearer expectations when compared to other STEM courses graded with a traditional grading scheme; however, there was no difference between specifications-graded and traditionally graded courses in student perceptions of the other three hypothesized student outcomes (i.e., reflect student learning outcomes, provide useful feedback, and promote motivation to learn). No other studies have empirically measured any of these outcomes.

### Empirical Investigations of Grade Outcomes

Most studies on the implementation of specifications grading in chemistry courses to date explore changes in the final course grade distribution. Specifically, researchers have investigated the effects on final grades before and after the transition to specifications grading.^21,34,40^ One team of researchers compared five semesters of final course grades in a traditional grading scheme with five semesters of grades in a specifications grading scheme in an introductory physical chemistry course with an average enrollment of 21 students.^21^ The authors report no statistical differences in the pre- and post-transition grade distributions and in the failure rates (i.e., rate of D, F, and W grades). Comparing grades for a graduate-level organic chemistry course over multiple semesters, researchers found increases in A grades with an overall 10% increase in passing grades.^40^ Another example in a large-enrollment organic chemistry laboratory also reports increases in A and B grades with corresponding decreases in the proportion of C and D grades with no changes in F grades.^34^

Not all stories of specifications grading implementations are as neutral or a success though. For example, in a second-semester general chemistry course with combined lecture and laboratory components, there was an overall redistribution of grades in switching to a hybrid specifications/traditional grading scheme from a fully traditional grading scheme.^30^ In both grading schemes, roughly 60% of students earned an A or a B grade; in the hybrid specifications/traditionally graded course, the number of A grades doubled, whereas the number of B grades halved compared to the fully traditionally graded course showing a redistribution of the A and B grades. Additionally, in this hybrid specifications/traditionally graded course, nearly 40% of students received a C, D, F, or W grade. While the percentage of C grades was lower in the hybrid specifications/traditionally graded course, there was an increase of nearly 7.5% in DFW grades.^30^ In a survey of a chemistry course, the proportion of students who withdrew from the traditionally graded course dramatically and worryingly increased from 16.5% (*n* = 115 of 695 students) to 35.4% (*n* = 22 of 62 students) under specifications grading.^41^ In a first-semester organic chemistry course at the same institution, the combined DFW rate alarmingly increased from 27.9% to 68.9% when switching from traditional (*n* = 77 of 276 students) to specifications grading (*n* = 62 of 90 students).^41^ However, it is important to note that these studies were conducted during the COVID-19 pandemic, which may have led to amplified effects.

### Empirical Investigations of Equity Outcomes

Proponents of specifications grading have also hypothesized that alternative grading schemes, such as specifications grading, support grade equity where there is a reduction in grade disparities between students of different social identities and backgrounds.^12,42–48^ To date, only one report has explored the extent to which specifications grading supports grade equity.^49^ In a large-enrollment (∼350 students) introductory child psychology course, researchers disaggregated the grade distribution by race (i.e., White, Asian, and underrepresented groups which includes American Indian/Alaska Native, Black, Hispanic, and Native Hawaiian/Pacific Islander identities) and first-generation versus continuing-generation status. In the traditional grading scheme, White and Asian students received A grades at a rate more than double the proportion of their underrepresented peers. However, in the specifications grading scheme, the proportion of underrepresented students receiving an A grade doubled, but the opportunity gap between these students and their White and Asian peers remained the same. It is noteworthy that the rate at which D, F, and W grades (i.e., DFW rate) were awarded in the specifications grading scheme was comparable to that in the traditional grading scheme: White and Asian students received fewer DFW grades than did their underrepresented peers. When comparing first-generation and continuing-generation students, systemically advantaged continuing-generation students received 18% more A grades in the traditional grading scheme than first-generation students, and this grade inequity expanded to 29% in the specifications grading scheme. Notably, the DFW rate quadrupled for first-generation students under the specifications grading implementation compared to traditional grading, whereas this rate merely doubled for continuing-generation students.^49^

### Research Question

In light of the findings present in the literature, it is crucial to examine the efficacy of specifications grading in yielding equitable grade outcomes.^37^ While previous studies primarily involve small-enrollment and/or upper-division courses, there is a lack of understanding the effects of transitioning to specifications grading particularly in a large-enrollment chemistry course. Additionally, no studies have explored equity in grade outcomes between student subgroups in traditionally graded and specifications-graded chemistry courses using an intersectionality approach. Therefore, the overarching question we aim to answer in this study is to what extent does specifications grading provide equitable grade outcomes in a large-enrollment general chemistry laboratory course?

## Methods

This study was conducted under Protocol no. 5362 as reviewed and approved by the University of Virginia Institutional Review Board (IRB) for the Social and Behavioral Sciences.

### Institutional Context

This study took place at a large public research university in the mid-Atlantic region of the United States. The university enrolls under 20,000 undergraduates annually. Approximately 56% are female and 17% are first-generation students. The institution is a predominantly white institution with under 20% of students identifying as U.S. residents of an ethnicity that is federally considered of underrepresented minority status (i.e., students who identify as American Indian or Alaska Native, Black or African American, Hispanic, and Native Hawaiian or other Pacific Islander, whether as a sole or one of multiple racial/ethnic categories). The largest colleges at the university, Arts and Sciences and Engineering, jointly enroll 83% of the undergraduate students. Most of the study participants are enrolled in one of these two colleges.

### Study Participants and Course Context

Study participants consisted of students enrolled in general chemistry laboratory courses in the academic years (AYs) from 2017 to 2019 and 2021 to 2023. The one-credit general chemistry laboratory courses include the Introductory College Chemistry I Laboratory, Introductory Chemistry I for Engineers Laboratory, Introductory College Chemistry II Laboratory, and Introductory Chemistry II for Engineers Laboratory. Courses in the AY17–18 and AY18–19 used a traditional grading scheme, whereas courses in the AY21–22 and AY22–23 used a specifications grading scheme.

Details regarding the specific implementation of this specifications grading scheme and the traditional implementation can be found in the Supporting Information. In brief, the differences between the traditional and specifications assignments in the courses can be generalized more as a reorganization of assignment categories rather than a complete change in the types of assignments. For example, scientific communication (one of the learning objectives) is its own assignment in the specifications-graded course rather than integrated into postlab assignments in the traditionally graded course. Course grades were determined by the number of learning objectives met in a bundling system (see the far left bundling model in Figure 1).

Two general chemistry I (GC1) laboratory courses were combined, as were the two general chemistry II (GC2) laboratory courses, because both course sequences were identical and only differed in composition of students’ major. The courses for engineering students were only offered pre-COVID-19 pandemic, and the two separate courses were combined in a single general chemistry laboratory course for all students post-COVID-19. GC1 Lab is only offered in the fall semester and typically enrolls 1600 to 1700 students, and GC2 Lab is only offered in the spring semester and typically enrolls 800–900 students. Students must be concurrently enrolled in or have completed the corresponding three-credit general chemistry lecture course. Students enrolled in the GC2 Lab in Spring 2022 or 2023 likely experienced specifications grading in the GC1 Lab.

### Course Grades

Final course grades were assigned using an institutional letter grading system. In this system, each letter grade corresponds to a numeric scale to calculate the grade point average (GPA). The letter-to-GPA conversions are as follows: A+ = 4.0, A = 4.0, A– = 3.7, B+ = 3.3, B = 3.0, B– = 2.7, C+ = 2.3, C = 2.0, C– = 1.7, D+ = 1.3, D = 1.0, D– = 0.7, and F = 0.0.

### Social Identities

Four social identities are used in this study: gender, generation status, underrepresented minority (URM) status, and transfer status. These social identities are self-reported by students as part of the college application process, except for transfer, which is designated by the institution. Student year is also reported (Table 1) to describe the sample but is not used in any analyses. Students’ social identities were obtained from institutional data per the approved IRB procedures.

**Table 1:** Participants’ Social Identities

	traditional grading		specifications grading	
	GC1 Lab (*n* = 3038)	GC2 Lab (*n* = 1673)	GC1 Lab (*n* = 3248)	GC2 Lab (*n* = 1759)
student year				
first	2471 (81.3%)	1166 (69.7%)	2721 (83.8%)	1245 (70.8%)
second	473 (15.5%)	406 (24.3%)	433 (13.3%)	413 (23.5%)
third	73 (2.4%)	71 (4.2%)	67 (2.1%)	72 (4.1%)
fourth	23 (0.8%)	30 (1.8%)	27 (0.8%)	29 (1.6%)
gender				
male	1393 (45.9%)	565 (33.8%)	1440 (44.3%)	471 (26.8%)
female	1645 (54.1%)	1108 (66.2%)	1808 (55.7%)	1288 (73.2%)
generation status				
continuing-generation	2676 (88.1%)	1489 (89.0%)	2765 (85.1%)	1515 (86.1%)
first-generation	362 (11.9%)	184 (11.0%)	483 (14.9%)	244 (13.9%)
URM Status				
not URM	2603 (85.7%)	1445 (86.4%)	2753 (84.8%)	1479 (84.1%)
URM	435 (14.3%)	228 (13.6%)	495 (15.2%)	280 (15.9%)
transfer status				
first-year admit	2977 (98.0%)	1617 (96.7%)	3190 (98.2%)	1693 (96.2%)
transfer	61 (2.0%)	56 (3.3%)	58 (1.8%)	66 (3.8%)

#### Gender

Institutional data include a binary gender classification. The terminology associated with binary gender at this institution is female and male.

#### Generation Status

First-generation students are defined as students from families in which no parent (or guardian) has obtained a four-year college degree. This definition aligns with the definition for first-generation students described by the Center for First-Generation Student Success.^50^ We regard students who do not identify as first-generation students as “continuing-generation students” in this study.

#### URM Status

A student is part of the URM group if they are a U.S. resident of an ethnicity that is federally considered to have underrepresented minority status: American Indian or Alaska Native, Black or African American, Hispanic, and Native Hawaiian or other Pacific Islander. Multiracial students are included in the URM category if one of their identities is considered underrepresented. International (i.e., nonresident) students are classified as non-URM.

#### Transfer Status

Transfer students include all students who were admitted with credit from another institution, while those who enroll directly from high school are considered first-year admits.

### Measure of Systemic Inequities

Intersectionality provides a framework to understand the sources of systemic inequities. Intersectionality deviates from the traditional framework to consider different identity dimensions (e.g., gender/sex, race) as mutually exclusive.^51^ Traditional frameworks for evaluating educational innovations have typically relied on analyses of singular social identity dimensions.^52–54^ We recognize that analyses using singular social identity dimensions are conducted in this study and have benefits for understanding the data along these single dimensions. However, singular identity analyses fail to account for the complexities that arise from intersections of these different dimensions^55–58^ in which students’ social identity affects their educational experiences.^59–61^

The systemic advantage index (SAI) is a metric that uses selected social identities to partially reflect systemic oppression in the U.S. higher education system through an intersectional approach.^62,63^ Researchers that first introduced this index have defined SAI as the number of advantages a student has based on selected demographic information; in that study, the researchers used students’ sex, race/ethnicity, income, and first- versus continuing-generation status to define SAI, in their context, which ranges 0–4.^62,63^

The selected social identities used in this study include gender, generation status, URM status, and transfer status. The SAI is therefore a measure of the number of advantages a student has based on these social identities and, in this context with these social identities, ranges 0–4. For example, a student with an SAI of 0 would be the least systemically advantaged and include first-generation, URM, female, and transfer students; a student with an SAI of 4 would be the most systemically advantaged and include continuing-generation, non-URM, male, and first-year admit students.

There are 16 mutually exclusive groups of students with different combinations of advantages (Table 2). Students within the same SAI have the same number of systemic advantages, but the exact advantages for students between groups differ (e.g., being non-URM versus being male for SAI = 1). While the SAI metric applies equal weight to each social identity, we acknowledge that the advantages are not equivalent.^62^ However, the SAI metric does facilitate how these advantages manifest in an intersectionality framework and provides more information about the interaction of these advantages over single measures of individual advantages alone.

**Table 2:** Participants’ Social Identities by a Systemic Advantage Index

		GC1 Lab		GC2 Lab	
SAI	description	Trad.	Specs.	Trad.	Specs.
0	first-generation, URM, female, transfer students	2 (0.07%)	1 (0.03%)	3 (0.18%)	3 (0.17%)
1	first-generation, non-URM, female, transfer students	65 (2.16%)	109 (3.39%)	40 (2.42%)	72 (4.11%)
	first-generation, URM, male, transfer students				
	continuing-generation, URM, female, transfer students				
	first-generation, URM, female, first-year admit students				
2	first-generation, non-URM, male, transfer students	396 (13.16%)	456 (14.17%)	249 (15.04%)	314 (17.93%)
	continuing-generation, non-URM, female, transfer students				
	first-generation, non-URM, female, first-year admit students				
	continuing-generation, URM, male, transfer students				
	first-generation, URM, male, first-year admit students				
	continuing-generation, URM, female, first-year admit students				
3	continuing-generation, non-URM, male, transfer students	1472 (48.94%)	1568 (48.73%)	931 (56.22%)	1011 (57.74%)
	first-generation, non-URM, male, first-year admit students				
	continuing-generation, non-URM, female, first-year admit students				
	continuing-generation, URM, male, first-year admit students				
4	continuing-generation, non-URM, male, first-year admit students	1073 (35.67%)	1084 (33.69%)	433 (26.15%)	351 (20.05%)

The overwhelming majority of students in this sample are systemically advantaged. Overall, 82.3% of students have three or all four systemic advantages (SAI = 3 or 4). Students with two systemic advantages (SAI = 2) sum to 14.7%; very few students have one systemic advantage (SAI = 1; 30%), and less than 0.1% of students have no systemic advantage (SAI = 0). Due to the very small number of students in the SAI = 0 group, the following analyses will focus on all other SAI groups (SAI = 1, 2, 3, and 4).

### Data Analysis

Statistical analyses were carried out in RStudio version 2023.12.1 running R version 4.3.3.^64^ The “rstatix”, “stats”, and “vcd” packages were used to perform statistical tests.^65,66^ SAI visualizations were generated using the “ggplot2” package.^67^

To assess variations of the grade distributions across subgroups by individual social identities, chi-square tests of independence, or Fisher’s exact tests for sparse data (i.e., transfer status), were conducted. For chi-square tests, Cramer’s V was calculated to determine the effect size, which is a measure of association between two variables; for two degrees of freedom, *V* = 0.07 is a small effect, *V* = 0.21 is a medium effect, and *V* = 0.35 is a large effect.^68^ To determine statistically significant differences between grade categories for the single social subgroups, post hoc Tukey tests with the Bonferroni correction were performed; Bonferroni-adjusted *p*-values for post hoc tests are reported.

To assess differences in mean course grades comparing traditional and specifications grading, Wilcoxon rank sum tests with continuity corrections were performed. The Wilcoxon test is the nonparametric version of a two-sample *t*-test to account for non-normally distributed data and approximated the data using a continuous distribution. Wilcoxon effect sizes (*r*) were calculated to determine the effect size, which is a measure of the magnitude of the effect as compared to random noise: *r* = 0.10–0.29 is a small effect, *r* = 0.30–0.49 is a moderate effect, and *r* ≥ 0.50 is a large effect.^68^

Binary logistic regression was used to model the probabilities of success in traditional and specifications grading based on students’ social identities. The outcome variable was binary high and low grade; high grades include A+, A, A–, B+, B, and B– grades, whereas low grades include C+, C, C–, D+, D, D–, F, and W grades; we include the C-range grades in the low grades as prior work has demonstrated that some students perceive a letter grade less than a B as an indication of failure,^69^ which in turn may lead students to not continue onto further chemistry courses. Predictor variables include gender (male = 0, female = 1), generation status (continuing-generation = 0, first-generation = 1), URM status (non-URM = 0, URM = 1), and transfer status (first-year admit = 0, transfer = 1). Variance inflation factors (VIFs) were below 1.12, indicating no multicollinearity between predictor variables. Probabilities and unstandardized odds ratios (ORs) are reported for each predictor variable. A *z*-score was calculated to test for the differences in odds ratios between traditional and specifications grading for each social identity.

## Results

The goal of this study is to empirically test the hypothesis that specifications grading supports grade equity in STEM-learning environments. We leverage grades and social identity data collected in large general chemistry laboratory courses before and after the implementation of specifications grading to test this hypothesis. We begin by reporting results using individual social identities (i.e., gender, URM status, first-generation status, transfer status), followed by results using intersectional identities represented by the SAI value.

### Grade Distributions

Students of all social identities received higher grades, specifically A grades, in the specifications-graded courses compared to the traditionally graded courses (Figures 2 and 3). For example, the proportion of first-generation students that received A grades in the GC1 Lab (Figure 2B) was 57.5% in the traditionally graded course and 77.9% in the specifications-graded course, an increase of 20%. For URM students in the GC1 Lab (Figure 2C), A grades were 26.2% higher after switching to specifications grading. These trends also hold for the GC2 Lab. For example, the proportion of A grades for URM students in the GC2 Lab (Figure 3C) had the largest difference of 37.2% (38.2% in traditional grading and 75.4% in specifications grading). Additionally, transfer students similarly proportionally earned higher grades in the GC2 Lab (Figure 3D) with 42.9% receiving A grades in traditional grading and 75.8% receiving specifications grading.

**Figure 2:**
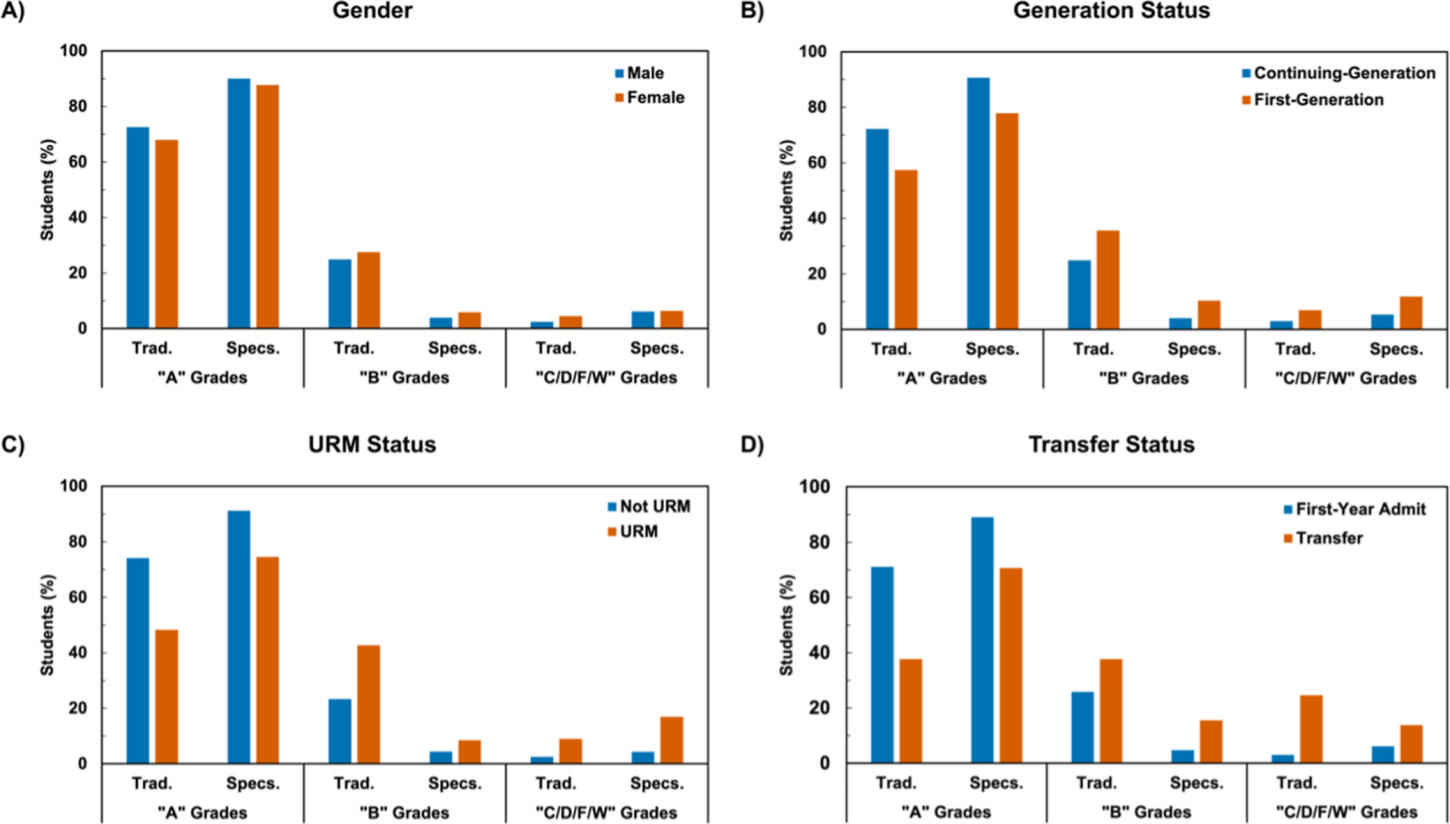
Course grade breakdown by a grade bundle between traditional and specifications grading for the GC1 Lab by (A) gender, (B) generation status, (C) URM status, and (D) transfer status. Trad. = traditional grading scheme. Specs. = specifications grading scheme.

**Figure 3:**
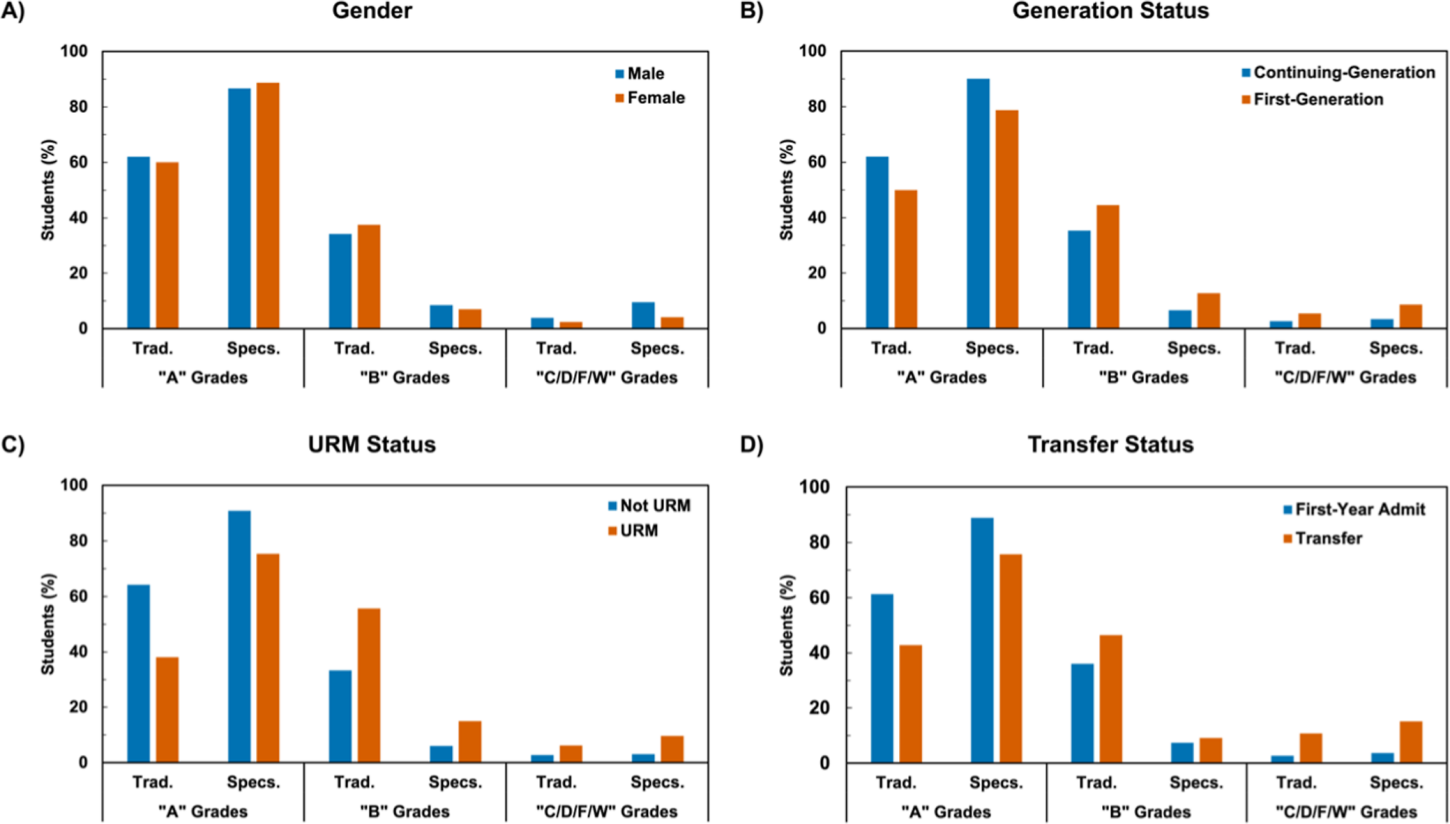
Course grade breakdown by the grade bundle between traditional and specifications grading for the GC2 Lab by (A) gender, (B) generation status, (C) URM status, and (D) transfer status. Trad. = traditional grading scheme. Specs. = specifications grading scheme.

However, nearly all groups of students also received a higher proportion of lower grades (i.e., C/D/F/W grades) in specifications-graded courses compared to traditionally graded courses in both GC1 and GC2 Laboratories. For example, in the GC1 Lab course, while the difference in lower grades was only slightly higher for males (3.6%) than females (1.9%) in the specifications-graded version of the course compared to the traditionally graded version, differences for first-generation (4.9%) and URM (8.0%) students were much larger than their systemically advantaged continuing-generation (2.4%) and non-URM (1.8%) counterparts. Interestingly, the lower grades were 3.2% higher for first-year admit students in the specifications-graded course compared to the traditionally graded course but were 10.8% lower for transfer students in the GC1 Lab (Figure 2D), while transfer students receiving lower grades were 4.4% higher in the specifications-graded GC2 Lab compared to the traditionally graded version (Figure 3D).

To determine whether the proportions of grade categories (i.e., A vs B vs C/D/F/W grades) differ across the four different subgroups of identities (e.g., male versus female), chi-square tests were conducted for gender, generation status, and URM status, and Fisher’s exact tests were conducted for transfer status. Fisher’s exact tests were used due to the small number of transfer students in our sample. Chi-square and Fisher’s exact tests indicate that the distributions of grades are statistically different between students of different generation, URM, and transfer status identities in both traditionally and specifications-graded versions of the GC1 Lab course (Table 3) and GC2 Lab course (Table 4).

**Table 3:** GC1 Lab Differences in Grade Distribution Based on Social Identities^a^

				differences within grade categories		
social identity	χ^2^	*p*-value	Cramer’s V	A	B	C/D/F/W
traditional grading (*n* = 3038)						
gender	13.44	0.001	0.07 (small)	√		√
generation status	38.59	<0.001	0.05 (small)	√	√	√
URM status	134.64	<0.001	0.21 (medium)	√	√	√
transfer status		<0.001		√		√
specifications grading (*n* = 3248)						
gender	6.85	0.033	0.11 (small)		√	
generation status	67.84	<0.001	0.15 (small-medium)	√	√	√
URM status	133.80	<0.001	0.21 (medium)	√	√	√
transfer status		<0.001		√	√	

a Note: √ marks indicate that the post hoc tests are statistically significant between groups for that grade category (i.e., a difference between males and females for the gender category).

**Table 4:** GC2 Lab Differences in Grade Distribution Based on Social Identities^a^

				differences within grade categories		
social identity	χ^2^(2)	*p*-value	Cramer’s V	A	B	C/D/F/W
traditional grading (*n* = 1673)						
gender	4.12	0.128	0.05 (small)			
generation status	12.10	0.002	0.09 (small)	√		
URM status	58.36	<0.001	0.19 (medium)	√	√	√
transfer status		0.001		√		√
specifications grading (*n* = 1759)						
gender	1.10	0.576	0.03 (small)			
generation status	27.67	<0.001	0.13 (small)	√	√	√
URM status	57.03	<0.001	0.18 (medium)	√	√	√
transfer status		<0.001		√		√

a Note: √ marks indicate that the post hoc tests are statistically significant between groups for that grade category (i.e., a difference between first-generation and continuing-generation for the generation status category).

In the GC1 Lab course, there are statistically significant differences in the distribution of grades among students of all social identities under both grading schemes (Table 3). Cramer’s V effect sizes indicate small and medium associations between the social identities and course grade under traditional grading. When comparing the effect sizes between traditional and specifications grading, these effect sizes remain relatively unchanged or increase slightly, suggesting that the association between social identities and course grade is not reduced in specifications grading and thus that grade inequities are still present.

In the GC2 Lab course, there are statistically significant differences in the distribution of grades for students of different generation, URM, and transfer status identities under both grading schemes (Table 4). Notably, there are no statistically significant differences in the grade distribution between males and females in both traditional and specifications grading. However, Cramer’s V effect sizes also indicate small and medium associations between all social identities and course grade. Once again, these results show little-to-no change in the effect sizes when comparing specifications grading to traditional grading, which indicates little change in improving grade equity.

In summary, the results of these analyses from both GC1 and GC2 Lab courses suggest that specifications grading is not improving grade equity but also not amplifying grade inequities at the single social identity level.

### Modeling Student Success

Binary logistic regression models were used to explain the association of the four individual social identity groups with the final course grade. High vs low grades are modeled where high grades represent A and B level grades, and low grades represent C and lower grades as a proxy for success in the course. The four social identity groups are used as predictor variables in the models: gender, generation status, URM status, and transfer status. For each, the systemically advantaged group is the reference group in the model (i.e., 0) and the systemically disadvantaged group is the group of interest (i.e., 1); for example, male is 0 and female is 1. In total, four logistic regression models were performed, one for each of the two grading schemes in each of the two courses. The results of the logistic regression models are shown in Table 5.

**Table 5:** Logistic Regression Results Modeling Student Success Based on Social Identities^a^

		traditional grading				specifications grading				differences (Specs.–Trad.)		
course	social identity	OR	SE	*p*-value	*P* (%)	OR	SE	*p*-value	*P* (%)	ΔOR	*p*-value for ΔOR	Δ*P* (%)
GC1 lab	female	1.97	0.16	0.001	66.3	1.01	0.15	0.925	50.3	–0.96	0.003	–16.0
	first-gen.	0.55	0.21	0.017	35.4	0.65	0.18	0.014	39.3	0.10	0.546	3.9
	URM	0.27	0.22	<0.001	21.1	0.25	0.16	<0.001	19.9	–0.02	0.783	–1.2
	transfer	0.09	0.33	<0.001	8.1	0.38	0.40	0.014	27.3	0.29	0.005	19.2
GC2 lab	female	1.51	0.38	0.281	60.2	0.90	0.29	0.720	47.4	–0.61	0.281	–12.8
	first-gen.	0.39	0.45	0.036	28.1	0.47	0.29	0.008	32.0	0.08	0.724	3.9
	URM	0.31	0.42	0.005	23.7	0.38	0.27	<0.001	27.6	0.07	0.679	3.9
	transfer	0.21	0.59	0.010	17.7	0.30	0.46	0.009	23.0	0.09	0.660	5.3

a Note: OR = unstandardized odds ratio. SE = standard error. *P* = probability. First-gen. = First-generation. Specs. = Specifications Grading. Trad. = Traditional Grading.

Unstandardized odds ratios (ORs) represent the odds that an outcome will occur given a particular occurrence. In this study, ORs represent the odds of a course success (i.e., high course grade: A or B grade) when a student holds a certain social identity. ORs = 1 indicate no difference in odds between two groups (e.g., females and males), ORs > 1 indicate increased odds of course success between two groups (e.g., increased odds for females compared to males), and ORs < 1 indicate decreased odds of course success between two groups (e.g., decreased odds for females compared to males) when all other social identities are held constant. For example, in the GC1 Lab under traditional grading, the odds of female students succeeding in the course are 1.97 times higher than the odds for males when all other social identities are held constant, and this is statistically significant (*p* = 0.001). When ORs are less than one, the reciprocal of the OR can be taken to determine the odds for the reference group (i.e., the systemically advantaged group); for example, in the GC1 Lab under traditional grading, the odds of first-year admit students succeeding in the course are (1/0.09 =) 11.1 times higher than the odds for transfer students and this is also statistically significant (*p* < 0.001). Therefore, the smaller the OR (if the OR is smaller than 1), the greater the odds of course success for the systemically advantaged group (i.e., male, continuing-generation, non-URM, and first-year admit students) when compared to the systemically disadvantaged group.

All students with systemically disadvantaged identities, except female students, were statistically significantly less likely to succeed in both grading environments (Table 5). This is evidenced by OR values smaller than 1 which represent inequities within the social identity group. Moreover, substantial improvement in equitable outcomes was only achieved with respect to gender in both courses and transfer status in the GC1 Lab. The odds of female students succeeding in these courses were higher under the traditional grading scheme (OR = 1.97) but are more on par with those of male students under specifications grading (OR = 1.01). The statistically significant difference in these ORs (ΔOR = −0.96, *p* = 0.003) indicates that there is more grade equity in the GC1 Lab between male and female students under specifications grading. The odds of transfer students succeeding in the GC1 Lab, as compared to their first-year admit counterparts, increased substantially from the traditionally graded course (OR = 0.09) in the specifications-graded course (OR = 0.38). Indeed, the difference in these ORs is statistically significant (ΔOR = 0.29, *p* = 0.005), indicating that there is more equity in the GC1 Lab between transfer and first-year admit students under specifications grading. Despite this positive change in the gap in odds between transfer and first-year admit students, this gap in odds of success is still wide under specifications grading, as an OR of 1 would suggest equitable outcomes. Therefore, the data suggest that while there are more equitable outcomes for transfer students in specifications grading than in traditional grading, parity is not achieved. For the other social identities (i.e., first-generation and URM) in the GC1 Lab and all social identities in the GC2 Lab, there are no substantial changes in the odds ratios as indicated by nonstatistically significant *p*-values for ΔOR (Table 5), suggesting that specifications grading did not statistically significantly increase the odds of success in these general laboratory courses for students with these identities.

Probabilities (*P*) of course success (i.e., A or B grades) are provided for each of the systemically disadvantaged social identities. We offer this additional interpretation using probabilities in addition to OR because ORs are often misinterpreted.^70^ When considering transfer students alone and holding all other social identities constant, transfer students in traditional grading have the lowest probabilities of receiving an A or a B grade in both GC1 and GC2 Lab courses, at 8.1% and 17.7%, respectively. However, the probability for transfer students’ course success is much higher for the GC1 Lab using specifications grading, at 27.3%. Course success for first-generation students in both lab courses and URM students and transfer students in the GC2 Lab have less than 5% higher probability of receiving an A or a B in specifications-graded courses compared to traditionally graded courses. The decrease in probabilities for female students in the GC1 Lab, when considered in the context of the odds ratio, indicates more equitable grade outcomes between female and male students (OR = 1.01, *p* = 0.925). This trend was also true for the GC2 Lab; a decrease in probability, but there was also equity between males and females under both traditional (*p* = 0.281) and specifications grading (*p* = 0.720). Transfer students have the lowest probabilities for course success in traditional grading, but this group greatly benefits from specifications grading particularly in the first-semester course, with a 19.2% higher probability of earning an A or a B in specifications-graded courses than in traditionally graded courses. Conversely, there are minimal improvements in URM students earning an A or a B in the GC1 Lab for specifications grading compared to traditional grading.

Overall, findings suggest that specifications grading minimally improved the odds of first-generation and URM students to succeed in the course when compared with students in traditionally graded courses, but equity was still not achieved. Even though there was a notable increase in transfer students’ odds of succeeding in the GC1 Lab when specifications grading was implemented, it did not eliminate the success gap in comparison to first-year admit students. Therefore, these odd ratio analyses further suggest that the implementation of specifications grading minimally addressed equity gaps.

### Systemic Advantage Index

To capture a more realistic picture of the potential for specifications grading to address equitable outcomes, we applied an intersectionality lens to the data by exploring relationships between grades and the SAI index. This approach acknowledges how systemic inequities compound for students who navigate having multiple intersecting identities. Figure 4 shows average course grades for students in the traditional and specifications grading GC1 and GC2 laboratories in each of the SAI groups.

**Figure 4:**
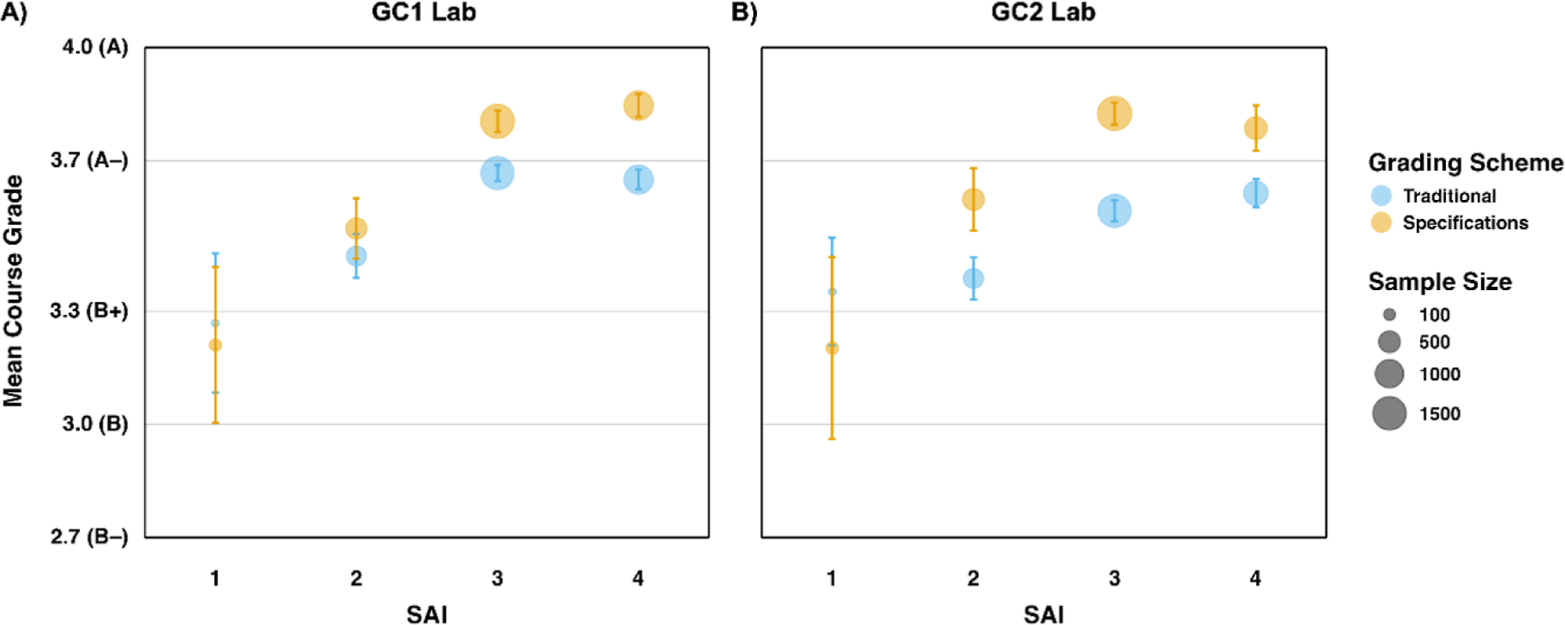
Average course grade point average by the systemic advantage index (SAI) for the (A) GC1 Lab and (B) GC2 Lab with traditional and specifications grading schemes. The sizes of the circles are proportional to the number of students in each group. 95% confidence intervals are shown. SAI = 0 is not shown due to very small sample sizes (*n* = 3 for the GC1 Lab and *n* = 6 for the GC2 Lab).

With one exception, students received higher grades in the specifications-graded GC1 and GC2 Lab courses (Figure 4, yellow dots) than in the traditionally graded version (Figure 4, blue dots). For example, on a 4-point scale, the average course GPA in the GC2 Lab improved from 3.39 (traditional) to 3.60 (specifications) for students holding two systemic advantages from their social identities (SAI = 2; Figure 4B). Therefore, specifications grading results in more systemically disadvantaged students, particularly students with SAI = 2, passing these courses with higher grades, which may contribute to their retention in STEM.

However, students that are the most systemically disadvantaged (SAI = 1) show a decrease in course grades when they switch to specifications grading (Figure 4), although there is a smaller sample size for this SAI group and a larger spread of grades. A Wilcoxon ranked sum test suggests that the mean grades are not statistically significantly different in the traditionally and specifications-graded courses in both the GC1 Lab (*W* = 3000, *p* = 0.083, r = 0.13) and GC2 Lab (*W* = 1256, *p* = 0.256, r = 0.11) for students with SAI = 1. Therefore, these data suggest that specifications grading may result in amplified inequities for the most systemically disadvantaged students as they are not receiving higher grades in specifications grading. Given the small proportion of students with SAI = 1, replication of this work at institutions with a more diverse group of students is necessary to test the viability of this trend.

Finally, we explored whether equitable outcomes were achieved within each grading setting. Kruskal–Wallis tests suggest statistically significant mean differences between SAI groups in all courses: the GC1 Lab with traditional grading (χ^2^(4, Ν = 3008) = 90.35, *p* < 0.001; Figure 4A, blue dots), GC1 Lab with specifications grading (χ^2^(4, Ν = 3218) = 220.11, *p* < 0.001; Figure 4A, yellow dots), GC2 Lab with traditional grading (χ^2^(4, Ν = 1656) = 64.70, *p* < 0.001; Figure 4B, blue dots), and GC2 Lab with specifications grading (χ^2^(4, Ν = 1751) = 138.96, *p* < 0.001; Figure 4B, yellow dots). These results indicate that both contexts (traditionally graded and specifications-graded) result in differential student outcomes across intersectional identities. However, post hoc Dunn’s tests indicate that the mean GPA between SAI = 3 and SAI = 4 for both types of grading in both courses is not statistically different (all *p* > 0.05); this means that there were no grade inequities between the most systemically advantaged students in traditional grading, and specifications grading did not change that. Moreover, looking specifically at the slopes of lines between SAI = 1 and SAI = 2, and between SAI = 2 and SAI = 3, for both traditional and specifications grading in the GC1 Lab (Figure 4A), ANCOVA tests indicate that the interaction effect between SAI and the grading scheme is not statistically significant (*p* = 0.336 and *p* = 0.144, respectively), meaning that the slopes between traditional and specifications grading are not different. For the GC2 Lab, an ANCOVA test for this interaction effect between SAI = 2 and SAI = 3 and the grading scheme is also not statistically significant (*p* = 0.320), which also suggests that the two slopes between SAI = 2 and SAI = 3 for both types of grading are not different from one another. Taken together, these results suggest that the inequities that primarily exist for systemically disadvantaged students in traditionally graded courses are also present in specifications-graded courses.

## Discussion and Implications

Specifications grading has gained great popularity among chemistry educators over the past decade despite little evidence of its effectiveness in addressing weaknesses of the traditional grading scheme. This study aims to empirically test the hypothesis that specifications grading supports equitable outcomes. Social identities and grades were collected from students enrolled in general chemistry laboratory courses under traditional and specific grading schemes. It is important to note that this study was conducted in a laboratory course, which may have implications different from those of a lecture-based implementation of specifications grading.

Grade distributions indicate that all students, including those holding systemically minoritized and disadvantaged social identities, pass these general chemistry laboratory courses with higher grades in specifications grading compared with traditional grading versions of the courses. This is promising, as receiving higher grades can help retain students in STEM. However, all three analyses conducted in this study demonstrate that specifications grading only minimally addresses equity gaps and that inequity between students of different identities present in the traditionally graded course persisted in the specifications-graded course. Our results are therefore conflicting. More systemically minoritized students pass specifications-graded courses with high grades, which can contribute to retention in STEM, but specifications grading did not close the opportunity gaps present in traditionally graded courses for minoritized students.

It is essential to note that the results of this study represent one type of implementation of specifications grading that closely follows the best practices advocated by Nilson. However, the literature on specifications grading in chemistry education details very different implementations even for the same type of course.^20,30,71^ Different implementations may lead to different outcomes. Therefore, we caution instructors, when implementing specifications grading, that their course and institutional context along with their implementation decisions may lead to results different from those in this study and different from those in the existing literature. Thus, we suggest and recommend that instructors who implement specifications grading engage in scholarship of teaching and learning (SoTL) projects to study the effects of specifications grading in their course. Centers of teaching and learning may offer support for these projects and may have a community of practice dedicated to alternative grading or SoTL projects. Additionally, we advocate instructors to form research–practice partnership with researchers who have expertise in educational research methods to empirically evaluate the impact of specifications grading.^72^

The results from this study point to the need to study at-scale the impact of specifications grading on student outcomes and to leverage these findings to conduct studies on the fidelity of implementation of specifications grading.^73^ These latter studies would help characterize the features of specifications grading implementations that minimize opportunity gaps in different learning environments. Importantly, these studies must characterize the social identities, attributes, and experiences of chemistry students who benefit and do not benefit under specifications grading to capture the link between implementations, student outcomes, and student experiences.

We are at a key moment in time with specifications grading. Early adopters are disseminating their instructional innovations through journal publications, conference presentations, and communities of practice resulting in an expansion of specifications grading use among committed instructors.^15,21^ Despite the limited empirical evidence of effectiveness for specifications grading, instructors readily connect with the philosophy of alternative grading and are quick to adopt alternative grading practices.^15^ This work showcases that specifications grading can potentially lead to systemically disadvantaged students’ retention in STEM. However, the implementations of specifications grading must be fine-tuned to optimize the expected outcomes, close opportunity gaps, and promote equity between students of differing social identities.

## Other Considerations

There are several considerations for this study. First, we, as researchers, defined what success in the course means. For our analyses, we defined any A or B range grade as a high final grade and any C, D, F, or W range grade as a low final grade. We have no understanding of students’ grade goals. In specifications grading, students can take advantage of the grading system and complete assignments to obtain their desired grade. This ground-up construction of a course grade allows students flexibility in balancing work for this course along with other coursework and other life obligations.^74^ For some students, a C grade may be the goal, because it fulfills their degree requirements.

Second, our findings are not intended to be generalizable to populations of students in different courses at the same institution or different institutions. Our findings relate to the specific population of students in the general chemistry laboratory courses using a specific implementation of specifications grading. We have made an effort to thoroughly describe our institutional and course contexts so that readers can understand how our context may have influenced our results and how findings may apply to the readers’ own context.

Third, our analyses lack control for potential confounding variables. For example, the data collected from the traditionally graded courses are pre-COVID-19 pandemic and the data collected from the specifications-graded courses are post-COVID-19 pandemic; therefore, the COVID-19 pandemic may have had an impact on students, such as on knowledge and grades, that we cannot account for. Additionally, many studies have reported that precourse GPA and standardized math scores (e.g., SAT and ACT) predict performance in general chemistry lecture courses;^75–78^ however, a recent study found that prior math preparation, as measured by students’ initial knowledge on the level of math involved on math-related chemistry questions, is not associated with the student learning rate in general chemistry lecture.^79^ Even if such data were collected, many institutions went test-optional as a result of the COVID-19 pandemic, including the study site, which leads to sparsity in reportable data. Moreover, lecture and laboratory courses are vastly different in content and goals, and thus, precourse GPA and standardized math scores may not predict performance in a laboratory-only course. Our study cannot also account for other variables such as prior laboratory experience or if students had previously experienced specifications grading or other kinds of alternative grading.

Fourth, the systemic advantage index is limited by its use of binary categories of social identities. For example, this binary classification is not inclusive of gender identities, including but not limited to, nonbinary and genderqueer students, and we recognize these data as a limitation of our study.^80^ Additionally, we recognize that URM status is a conglomeration of races and ethnicities and eclipses the nuances of identifying with specific races and ethnicities. We are limited to four social identities in this study because of our data source and note that privileges exist in other dimensions (e.g., income and ableism).^81–83^ However, this is the first study to include transfer status in the calculation of an SAI measure; previous studies use income in lieu of transfer status^62,63^ or leave out the income variable due to unavailability of that data.^84^ We recognize that students are more than just their gender, generation status, URM status, and transfer status and do not wish to invalidate the different aspects of students’ identities that cannot be captured in this study due to our data source.

## Conclusions

In this study, we analyzed the final course grades of over 9700 students in a year-long general chemistry course that used both traditional and specifications grading. Using three different quantitative approaches to understand student course grades, we find mixed results. On one end, more students with systemically disadvantaged identities are passing these courses with higher grades, which can lead to their retention in STEM majors and fields. On the other end, equity gaps in grades present in the traditional grading settings are only marginally addressed by specifications grading. Future research must characterize the features of specifications grading that minimize opportunity gaps and maximize student-focused outcomes under different learning environments as well as characterize the social identities, attributes, and experiences of chemistry students who benefit and do not benefit under specifications grading. The alternative grading movement is here to stay, and it is essential and timely to further evaluate and maximize the impacts of specifications grading.

## Supplementary Material









## Notes

GC1 Lab Syllabus using traditional grading (PDF)
GC2 Lab syllabus using traditional grading (PDF)
GC1 Lab syllabus using specifications grading (PDF)
GC2 Lab syllabus using specifications grading (PDF)
