# Supplementary material for: Balancing Equity in General Chemistry Laboratory Courses: The Complex Impact of Specifications Grading on Student Success and Opportunity Gaps
Source: JACS Au. 2025 May 19;5(6):2593–605. doi: 10.1021/jacsau.5c00210 (PMC12188485; doi:10.1021/jacsau.5c00210)
Supplement: Supplementary file 1 [file au5c00210_si_001.pdf]

[redacted]

[redacted] INTRODUCTORY CHEMISTRY I LABORATORY, FALL 2023

Welcome to General Chemistry Lab! In this course, we strive to create an environment where every student feels safe learning, sharing, and collaborating. Human diversity is welcome and celebrated in all its forms. If at any time you feel unwelcomed or unsupported in your learning, please reach out to your TA or the instructor.

This course is an introduction to experimental chemistry, developing laboratory skills and safety. Students plan and implement experiments in cooperative 4-person teams using a guided inquiry approach. Process skills include procedure development, data analysis, and scientific communication.

Table of Contents

1. THE WHEN AND THE WHERE 2

2. IMPORTANT CONTACTS 2

3. WHAT YOU'LL NEED 2

4. IMPORTANT DATES 3

5. WHAT'S A "WORKSHOP"? HOW IS IT DIFFERENT THAN A "LAB"? 3

6. SKILLS YOU'LL DEVELOP (STUDENT LEARNING GOALS) 4

7. THINGS YOU WILL DO TO DEVELOP THOSE SKILLS 4

8. WHAT IS ALL THIS ABOUT "MASTERED" AND "NOT YET MASTERED" AND TOKENS? 7

9. TIPS FOR SUCCESS 8

10. SAFETY 9

11. ATTENDANCE AND MAKE-UP OPPORTUNITIES 9

12. RE-GRADES 10

13. HONOR CODE 10

14. EXTRA CREDIT 11

15. ACADEMIC CONCERNS 11

16. STUDENTS WITH DISABILITIES 11

17. WHAT IF ALL COURSES [redacted] MUST TRANSITION ONLINE? 12

18. PERMISSION TO USE WORK AS FUTURE TRAINING TOOLS 12

19. STUDENT GRADE TRACKER 12

1. The When and the Where

Laboratory Times: [redacted]

Physical Laboratory Locations: [redacted]

Attendance is mandatory at all laboratory and workshop meetings. See the schedules and room assignments on [redacted]

Credit Hours: 1
Course Co-requisite: [redacted] must be taken with or prior to this course.

2. Important Contacts

[redacted]

[redacted]

Teaching Assistants: See [redacted] for TA names, their student hours, and how to attend. TA student hours are held in [redacted] (the large, open study space).

A Note About Correspondence Due to the large number of students enrolled in the course, we suggest you take any questions or concerns to your section TA first. If an answer or solution cannot be found, then contact the Head TA. If an answer or solution is still elusive, then contact [redacted].

3. What You'll Need

Physical Materials

1. Laptop: You need to have a personal laptop for this course. The software and computational tools we will use can be accessed on a phone or tablet, but are much, much easier to use on a laptop. Instructions for using these tools will be provided assuming laptop, not phone, use. As a student, you have access to a free storage account provided by Box. It is highly recommended that you use your Box account to back up your assignments in case of computer issues.
2. Notebook: "Introductory Chemistry Laboratory" notebook for [redacted], available in the bookstore. Used lab notebooks from other students are not allowed. No other notebooks allowed.
3. Safety Goggles: You can buy these at the bookstore, from the Chemistry Graduate Student Council or 3M tech safety goggles sold by local retailers as "3M Clear Plastic Chemical Impact Goggle". No other models allowed.
4. Lab coat: Available at the bookstore or the Chemistry Graduate Student Council
5. Calculator (with log and ln functions; graphing not necessary)

Websites and Software

1. [redacted] will be your place to view this syllabus, schedules, announcements, and lots of other important information. Check it regularly. It is possible to turn off email notifications for various things in [redacted] **Do not turn off email notifications for announcements!** [redacted] **announcements are the primary way I communicate with you and email notifications send those announcements right to your inbox.** A mobile app for [redacted] is also available if you prefer using your phone. Visit the App Store or Google Play and search for [redacted] To connect your app to [redacted], tap "Find my School" and search for [redacted]
2. LoggerPro: This software is free for students to download. Instructions will be given about how to do this before the first time LoggerPro is needed.
3. Gradescope: This service is free for students. It will be our system for uploading assignments, providing feedback, and releasing grades. Instructions for joining your section's Gradescope course will be provided at the beginning of the semester.

Suggested Reading

This resource is in no way required, but may help you develop your scientific writing skills. If you plan on doing future scholarly writing in Chemistry, you may wish to invest in access to the full guide.

The ACS Guide to Scholarly Communication. https://pubs.acs.org/doi/book/10.1021/acsguide

4. Important Dates

[redacted]

[redacted]

5. What's a "Workshop"? How is it Different than a "Lab"?

We have access to 8 laboratory rooms in the Chemistry building. To offer enough seats in the course, however, there are days and times when more than 8 sections must be offered concurrently.

Our solution to that problem is a system of alternating weeks of hands-on lab time and workshops. During lab weeks, you will meet with your section in one of the laboratory rooms [redacted] and implement your group's experimental design. During workshop weeks, your will meet with your section in a different room to develop the experimental design you plan to implement the following week.

Odd numbered sections (section numbers ending in 1, 3, 5, 7, or 9) will begin with a lab week during the second week of the semester [redacted]. Odd sections will end with a lab week [redacted] and be finished with formal class meetings just before the Thanksgiving break.

Even numbered sections (section numbers ending in 2, 4, 6, 8, or 0) will wait an additional week to begin, with a lab week during the third week of the semester [redacted] Even sections will continue after Thanksgiving break, having the last formal class meeting with a lab the week of [redacted]

See the full odd- and even- schedules on [redacted] Print out a copy and post it where you will see it often!

6. Skills You'll Develop (Student Learning Goals)

1. Complete background research and learning to fully understand the needs of an experimental problem.
2. Engage in experimental design, choosing appropriate laboratory techniques and equipment.
3. Implement experiments using common chemical laboratory techniques, good laboratory hygiene, appropriate safety precautions, and appropriate waste disposal.
4. Keep a laboratory notebook using standard notebook format and etiquette.
5. Organize, analyze, and draw conclusions from data.
6. Effectively communicate experimental ideas and chemical principles orally and in writing
7. Work collaboratively with peers.
8. Demonstrate individual understanding of techniques used in lab and chemical principles underlying the experiments completed.

7. Things You Will Do to Develop those Skills

<u>Prelab assignments</u>: 6 prelabs, of which the first one cannot be skipped

For each project, you will complete an individual pre-lab assignment to help prepare you for working with your team to develop an experimental plan. The prelab assignments will be submitted on Gradescope. The first prelab of the semester must be completed. A token can be used to turn it in late, if needed. Most prelabs will require between 1 and 2 hours to complete for most students.

Prelab assignments give you practice and feedback on Learning Goal 1 in Section 6.

<u>Experimental Plans and Summaries</u>: 5 plans, 6 summaries

You will work in a team to plan out your experiment and summarize each experiment. The purpose of planning and summarizing experimentation is to get you thinking systematically about how you approach solving a scientific question. Working on plans and summaries during class time will provide you opportunities to engage in teamwork, a valuable skill for virtually any career.

Every group must submit either a plan or a summary every week. Since they are submitted during class time, tokens also cannot be used to turn them in late. Individual group members are not expected to contribute to a plan or summary if they are absent. For example, if you are sick one week, you do not need to contribute to the group submission, but it may be wise to make up the missed work individually using a token, depending on which grade you are targeting and whether you are on track for that grade. If you plan to make up the missed work you must communicate with your TA so that it counts as an excused absence. If you miss lab/workshop for an unexcused reason you will receive a "Not Yet

Mastered" on any work done that day, and you will not be allowed to make up or resubmit the plan/summary.

Plans give you practice and feedback on Learning Goal 2 in Section 6. Summaries give you practice and feedback on Learning Goal 4 in Section 6.

Safety: Assessed by TAs every experimental week

Each day you are in the laboratory, your TA will assign you a Safety score of either Mastered (M) or Not Yet Mastered (NYM). To earn an "M" you must wear appropriate PPE, follow appropriate disposal instructions for chemical waste, and generally be a good laboratory citizen. This includes following all instructions from your TAs, instructors, and lab staff. If you forget to bring your lab coat, goggles, or wear appropriate shoes one day, you can still earn an "M" by quickly returning to your dorm/home, getting your things, and returning to lab. You will only earn "NYM" for not having appropriate dress if you try to argue with your TA or otherwise push back against wearing what you need to. You may also be assigned "NYM" if you need multiple reminders from your TA or an instructor about keeping your PPE on (e.g. having goggles up on your forehead instead of over your eyes).

Because laboratory safety is so important, there is no way to use a token to revise a safety score after it is assigned.

Safety Scores give you practice and feedback on Learning Goal 3 in Section 6.

Postlab assignments: 6 postlabs, of which the first one cannot be skipped

Post-lab assignments will be one way you will analyze your projects' findings and will help you make connections between your experimental approach and chemical principles. All post-lab assignments will be turned in individually on Gradescope. The first postlab of the semester must be completed. A token can be used to turn it in late. Most students will require between 1 and 2 hours to complete postlabs.

The last postlab of the semester **cannot** be revised and resubmitted using a token. This is due to the time needed to address resubmissions and our need to meet the Registrar's deadline for submission of final course grades.

Postlab assignments give you practice and feedback on Learning Goal 5 in Section 6.

Communicating Science: 1 presentation, 1 poster, 0-2 written assignments

Each team will present the results of their experimentation to their TA and lab section. The purpose of these presentations is to practice your public speaking and hone your ability to explain chemical phenomena to your peers. Following the presentations, your TA will hold a whole-class discussion about each team's approach to the project and help you make connections between your project, chemistry, and other real-world applications. Presentations cannot be made up. Components of presentations that take place "live", such as good oral delivery and participation, cannot be revised/resubmitted, but the other portions, such as slide contents, can be revised/resubmitted with the use of a token. Your group will present once this semester. You cannot choose to opt-out of the presentation, even if it is not needed for your target grade in the course, because your group mates need your help and participation.

Each team will also practice communicating results through a scientific poster, which is a common way scientists share their work with the scientific community. TAs will facilitate a poster session. Components of poster sessions that take place "live", such as asking and answering questions, cannot be revised/resubmitted, but the other portions, such as poster content, can be revised/resubmitted with the use of a token. Your group will prepare one poster this semester. You cannot choose to opt-out of the poster, even if it is not needed for your target grade in the course, because your group mates need your help and participation.

Some higher-level grades in this course require completion of scientific writing assignments. A token may be used to revise and resubmit if you do not achieve mastery on your first attempt. If you do not wish to earn these higher grades grade, you do not need to complete the writing assignments! Writing assignments are completed by individuals, not groups.

Use of AI to complete any scientific communication assignment is prohibited unless you are specifically instructed to do so. Assignments give you practice and feedback on Learning Goal 6 in Section 6.

<u>Peer Assessments</u>: 3 evaluations

You will be working in a team every week in lab or workshop, and you will have the opportunity to provide feedback and communicate to your team members on your perceptions of the team dynamics in your group. The purpose of providing feedback to students on their teamwork is to help students learn and improve on their skills in collaborative work.

Because it is impossible to re-do a teamwork scenario, Peer Assessments cannot be revised/resubmitted. Peer Assessments give you practice and feedback on Learning Goal 7 in Section 6.

<u>Content Knowledge Quizzes</u>: 3

Three times during the semester you will take a quiz to assess your understanding of chemical principles and laboratory techniques related to the most recent project(s). These multiple-choice and short-answer quizzes will be completed individually on Gradescope. Although they are open-book, you may not consult any humans for help, including anonymous humans on online forums or help pages, even if the online posts were written in the past. Use of AI is not allowed on quizzes.

Although quizzes are also assessed on a Mastered/Not Yet Mastered basis, they do not follow the same revise-and-resubmit rules as other assignment types. If you score Not Yet Mastered on a quiz, you can choose to spend a token to do a Try-Again Quiz. Try-again quizzes will be alternate versions of the original quiz. They will contain questions similar, but not identical, to the questions on the original quiz. If you choose to do the Try-Again Quiz, you are strongly encouraged to study the problems you missed the first time, and understand the process needed to answer each question. You are also encouraged to visit a TA's Help Center Hour with any questions that still confuse you. Each quiz will have only one Try-Again opportunity. If you do not Master the Try-Again Quiz, you are stuck with a "Not Yet Mastered" score for that quiz.

Quizzes give you practice and feedback on Learning Goal 8 in Section 6.

8. What is all this about “Mastered” and “Not Yet Mastered” and Tokens?

There will be five projects throughout the semester that encompass a variety of different activities and assignments that directly map to the course learning goals in Section 6. TAs assess all assignments against a standardized rubric. Rubrics will be available to you on [redacted] before assignments are due, to give you a better idea of what is expected and to improve transparency.

You may be familiar with grading structures based on the accumulation of points. In this course there are no points. Instead, a grading system called “specifications grading” will be used. In this system, only two grades are possible on any assignment: “Mastered” and “Not Yet Mastered”. Benefits and highlights of this system include:

- With only two possible outcomes, differences in grading between TAs will be minimized.
- You never need to stress about getting that last point to tip your grade from one grade to the next!
- Flexibility. You don’t have to do every assignment to get an A in the course.
- Embracing a Growth Mindset. There will be opportunities to learn from feedback on assignments where you get a “Not Yet Mastered”, revise your work, and resubmit. This will work using a Token system, which will be explained later.
- Rubrics are more straightforward, with only one category to strive for: Mastery.
- “Mastery” doesn’t mean perfection! You don’t have to achieve Mastery on every rubric item to get an overall grade of “Mastered” on an assignment.

<u>How does specifications grading work?</u>

A set of guidelines for each letter grade is outlined in the Student Grade Tracker handout posted on [redacted] and summarized at the end of this syllabus. To earn a letter grade, complete all the requirements listed for that grade.

Your final grade in the course is the highest category for which you meet **all** criteria. If you meet most of the requirements for a grade, but not all of them, you do not earn that grade. You earn the highest grade for which you meet all of the minimum requirements.

<u>What does “Mastery” mean?</u>

Earning a Mastered grade on an assignment means that you have met or exceeded the minimum requirements to show you have learned the material covered by that assignment. The exact requirements vary by assignment and are always listed on the rubric. The threshold for Mastering an assignment is usually satisfying 80% or more of individual rubric items, but this may vary from one assignment to another. As you can see by the 80% threshold, you can make mistakes and still achieve mastery, but you should aim to make your assignment submissions the best possible quality.

<u>What happens if I earn “NYM” (Not Yet Mastered) on an assignment?</u>

Generally, you have two options if you earn a “NYM”. If you have a token available to use, you can revise and resubmit your assignment *within one week of receiving your “NYM” score*. You only need to revise sections that were marked as “NYM” on the rubric. The last postlab of the semester (Solutions and

Spectroscopy Postlab) cannot be revised and resubmitted using a token. This is due to the time needed to address resubmissions and our need to meet the Registrar's deadline for submission of final course grades.

Or you may choose to accept your "NYM" score. This is an option because you do not need to earn a "M" score on every assignment in the class! Be sure the check the Student Grade Tracker for details on how many "M" scores are required in each category to earn each letter grade before choosing whether to revise/resubmit or to strategically accept the "NYM" grade.

Late Assignments

Late work will not be accepted, except if you "buy" that opportunity by using a token.

If you find that you are sick or otherwise facing a short-term problem stopping you from getting work done on time, you can choose to either use a token and submit that work late, or you may choose to strategically not do that assignment at all, because you don't need to get an "M" on every assignment to get an A in this course!

Tokens for late work are meant to be for short extensions, up to 72 hours after the original deadline. If you experience a longer-term problem needing more than 72 extra hours to complete work, including but not limited to prolonged illness or a documented disability [redacted], please contact the instructor (lnm2a) to discuss modifications to this policy and/or set up a plan to get or keep you on track.

Tokens

Tokens are a form of digital currency. They are your safety net and your way to handle things when "life happens". When you complete the "Understanding Grading in [redacted]" assignment, you will automatically earn 6 tokens. You can exchange tokens for various things in the class such as turning in an assignment late or a resubmission to attempt to turn a "NYM" into an "M" score. A detailed list of things which can be purchased with tokens will be posted on [redacted]

To use a token, you must write an e-mail to your grading TA, and cc Head TA [redacted], stating that you wish to do so and attaching your late or revised work. Your grading TA will then approve your use of a token, update your remaining token total in Gradescope, and give you any more instructions that are needed. Any dishonesty about how many tokens you have remaining will be treated as an Honor Code violation.

9. Tips for Success

You are expected to take responsibility for your own learning and be an active participant in all aspects of the course. This can sometimes seem intense or feel overwhelming. Below are some of the ways we have found students can prepare for and be successful in this course.

- Do background research related to the planning questions before coming to lab.
- Be an active participant in lab during planning, experimenting, and presenting.
- Wear appropriate attire during lab: long pants, socks, closed-toed shoes, lab coat, goggles.

- Prepare to do about 3 hours of work outside of lab each week on average. Some weeks will require more out-of-class work, and some will require less.
- Work on assignments ahead of the due date so you can attend Student Hours and get help when needed.
- Try your best to not get in your own way! It can be hard to speak up when things aren't going as well as you want. A common consequence of this silence is not learning as much as you could have. Your instructor and TAs are here to help, but we can't do that if we don't know what you are confused about.

It is very difficult to succeed if you do not understand the expectations of the course. If you are confused about what you need to do, just ask! Questions about what you should be doing before, during, and after lab are always welcome.

10. Safety

Safety is the most important goal of every laboratory experiment! Never complete an experimental step if you do not know what you are doing, why you are doing it, and how to minimize any risks involved. When in doubt, ask!

Safety rules must be always observed in this laboratory course. Any of the laboratory staff can immediately dismiss a student who poses a danger to themself or to anyone else. This includes proper attire and behavior. A student who is unwilling or unable to follow directions from an instructor or TA also poses a safety hazard and may be dismissed. A student who is unwilling to follow any necessary health precautions poses a health hazard and may be dismissed.

Adherence to safety procedures reduces the risk of accidents and injuries. However, if an accident or injury occurs, it must be reported immediately to a TA or lab supervisor. Cleanliness/tidiness and proper disposal of leftover chemicals are part of good safety practices.

11. Attendance and Make-up Opportunities

Since you will be working in collaborative teams during lab, it is essential you show up to lab on time and do not miss any class meetings. There is no make-up week for missed work. Should you miss an experiment for an excused reason, you will have to complete the required assignments (prelab, postlab, etc.) independent of your group and you are responsible for gathering any needed information (such as data) from your lab group. There are certain assignments that cannot be made up, even for an excused absence. The following constitute potentially excused absences: family emergency, death in the family, severe individual illness, being placed in isolation or quarantine, and participation on a [redacted] varsity athletic team that requires you to travel during your lab meeting. Your TA and lab group members must be notified by e-mail of the absence prior to lab unless it is due to a completely unforeseeable reason, in which case, you will have 48 hours after the missed lab to notify the instructor or TA. If you missed lab for any unexcused reason you will receive a "Not Yet Mastered" on any work done that day, and you will not be allowed to use a token to make up or resubmit the work.

Since there is no opportunity to make-up experimental work, and since laboratory courses are inherently experiential in nature, if you miss more than 2 class meetings (both in-person experiments

and workshops count as meetings), regardless of whether those absences were excused or unexcused, you may be asked to withdraw from the course. If the withdraw date has passed, you will receive a failing grade. *The minimum required attendance is 9 out of 11 possible weeks.*

12. Re-grades

If at any time you feel that any of your work was graded incorrectly, please don't hesitate to bring it to our attention by using the following protocol:

1. In Gradescope, navigate to the assignment in question and use the "Request Regrade" button. Be sure to explain (politely, and with complete sentences!) what work you would like to be reassessed, and the reason why you think the original grade was incorrect.
2. Your regrade request will be processed in a timely manner (target: less than one week). You may also be e-mailed if it is necessary to clarify something in your note.

Regrades will never be decided while the student involved is present. That creates unfair pressure on TAs or instructors to make a quick decision. Also remember to not make any changes to the assignment itself before requesting a regrade. Any change will be considered a violation of academic integrity and will be penalized accordingly. Any time you ask for a re-grade on completed work, we reserve the right to regrade any portion of that assignment, not just the part requested.

Requests for regrades must be submitted within one week of receiving your score on any assignment; no requests for regrades will be humored after that deadline. A regrade cannot be requested on the last postlab of the semester (Solutions and Spectroscopy Postlab). This is due to the time needed to address regrade requests and our need to meet the Registrar's deadline for submission of final course grades.

13. Honor Code

Because we want all students to learn to be more like scientists, whose reputations depend upon the trustworthiness of their work, the Honor System is taken very seriously in this course. By writing or agreeing to the pledge on your work, you are stating: [redacted] Do not hesitate to ask the TAs or your instructor for assistance throughout the semester.

In this course, academic dishonesty includes "fudging" laboratory data such as weights, melting points, etc. The fabrication of scientific data and information is an egregious violation of the underpinnings of scientific inquiry and the scientific method. Use of AI in completion of assignments is never allowed unless the assignment specifically asks students to use AI.

Any violation of this policy will be treated as outlined below.

<u>Penalties for Academic Integrity Violations</u>

Any instance of academic dishonesty (lying, cheating or plagiarism) will result in a grade of "Not Yet Mastered" on the experiment/assignment during which the dishonesty took place, and a token may

NOT be used to resubmit the work. Instances of dishonesty may be reported to the Honor Committee at the discretion of any student, TA, or instructor.

When it comes to plagiarism, it does not matter if you plagiarized or if you knowingly allowed someone to plagiarize off of you, the penalty is the same. When it comes to cheating, it does not matter if you cheated or if you knowingly allowed someone to cheat off of you, the penalty is the same.

14. Extra Credit

There is none. Please do not ask. Focus on strategic use of your tokens and doing quality work on remaining assignments.

15. Academic Concerns

Students are encouraged to discuss concerns about academic issues with faculty members according to the following sequence. If the concern is about a grade, be advised that it is your responsibility to keep all graded papers in the event that there is a discrepancy at the end of the semester.

1. A student should informally discuss any concerns with the instructor or TA involved. In most cases, this discussion should result in the clarification of any misunderstandings and/or a satisfactory resolution for all parties.
2. If a student is not satisfied with the outcome of this informal process, they should formally present their concerns in writing to an instructor (e-mail preferred).
3. A meeting between the student and instructor will be organized to discuss these concerns.
4. Following this meeting, the instructor will carefully consider the situation and present a decision in writing to the student.
5. If the student is not satisfied with the outcome of this formal process, they should consult with the Department Chair.

Students who skip steps in this sequence will probably not be satisfied with the outcome of their action.

16. Students with Disabilities

All students with special needs requiring accommodations should forward the appropriate paper- work from the [redacted]. It is the student's responsibility to present or forward this paperwork in a timely fashion and follow up with the instructor about the accommodations being offered. Accommodations for quiz-taking (e.g., extended time) should be arranged at least one week before a quiz.

If you are experiencing academic difficulties which you believe could be eased by having access to accommodations, please don't hesitate to reach out to [redacted]

17. What if all courses [redacted] must transition online?

If we transition online for only a short time, we will complete assignments using experimental data collected by TAs until we can transition back to in-lab experiments. If we transition online for a long time, supplemental software may be required to allow you to complete experimentation virtually. Course meetings will be conducted online, and instructions for attending will be disseminated as quickly as possible following the University's switch to online learning.

In any case of online transitioning, some modification of the grading scheme may be necessary. Any modifications to the schedule of experiments and/or grading policies will be communicated clearly as soon as possible after it is clear they are needed, using Collab Announcements.

18. Permission to Use Work as Future Training Tools

Whenever you submit an assignment in this course, you agree that it may be used as an example of student work during training/orientation of future graduate and undergraduate TAs. If your work is used in this way, it will be de-identified.

19. Student Grade Tracker

Students will earn the highest grade for which they meet all minimum requirements, as given in the table below. If you meet most of the requirements for a grade, but not all of them, you do not earn that grade. See next page for requirements for +/- grades. Students who do not meet the minimum requirements for a D- will be assigned a grade of F.

	A	B	C	D
Prelabs with 'M' grade (Learning Goal 1)	≥ 5	≥ 5	≥ 4	≥ 3
Plans with 'M' grade (Learning Goal 2)	≥ 4	≥ 4	≥ 3	≥ 3
Safety Assessments with 'NYM' grade (Learning Goal 3)	0	≤ 1	≤ 2	≤ 3
Summaries with 'M' grade (Learning Goal 4)	≥ 5	≥ 5	≥ 4	≥ 3
Postlabs with 'M' grade (Learning Goal 5)	≥ 5	≥ 5	≥ 4	≥ 3
Scientific Communication (Learning Goal 6)	Participation in 2 presentations, 4 'M' grades in combined presentations/writing	Participation in 2 presentations, 3 'M' grades in combined presentations/writing	Participation in 2 presentations, 2 'M' grades in combined presentations/writing	Participation in 2 presentations, 1 'M' grade in combined presentations/writing
Peer Assessments with 'M' grade (Learning Goal 7)	≥ 2	≥ 2	≥ 2	≥ 1
Quizzes with 'M' grade (Learning Goal 8)	3	≥ 2	≥ 1	≥ 0
Beginning of Term Activities (Safety trainings, software setup, Understanding Grading Assignment)	Complete all	Complete all	Complete all	Complete all
Attendance	At least 9 weeks OR individual plan developed with Instructor	At least 9 weeks OR individual plan developed with Instructor	At least 9 weeks OR individual plan developed with Instructor	At least 9 weeks OR individual plan developed with Instructor

Plus (+)	
One ‘M’ grade above the minimum in Prelabs, Postlabs, or Scientific Communication	AND Peer Assessment greater than minimum by 1

Minus (-)
Missed the minimum criteria for the full letter grade by just one Mastery grade in just one category

Token Exchange

Tokens may not be given or exchanged with other students. Your only transactional partner for tokens is your TA/instructor.

Things Tokens Cannot Be Used For:

- To Re-do or revise a Safety Score. Safety is too important.
- Take a quiz for the *first* time. If you miss a quiz for an excused reason (varsity travel, illness, family emergency, etc.) a makeup can be scheduled, no token needed.

Things Tokens Can Be Used For:

Cost	Item	Notes
1 token	Revise and Re-submit a NYM score	Can be used on prelabs, postlabs, plans, summaries, and scientific communication. Request must be received with revised work attached no later than one week after the NYM score is released. Only rubric items that earned NYM need to be revised.
1 token	Submit work after the due date	Can be used on prelabs, postlabs, plans, summaries, peer evaluations, and scientific communication. Request must be received with the late work attached no later than 72 hours after the due date. If an extension of more than 72 hours is needed, please contact [redacted] to devise an individual plan.
1 token	Take a Try-Again Quiz	Try-again quizzes will be alternate versions of the original quiz. They will contain questions similar, but not identical, to the questions on the original quiz. A try-again quiz cannot be taken if the original quiz was not taken.

How to Use Your Tokens:

Write an e-mail to your grading TA, and cc Administrative TA [redacted], stating that you wish to do so. **Include your late or revised work as an attachment.** Your TA will then approve your use of a token, update your remaining token total in Gradescope, and give you any more instructions that might be needed.
