# Supplementary material for: Balancing Equity in General Chemistry Laboratory Courses: The Complex Impact of Specifications Grading on Student Success and Opportunity Gaps
Source: JACS Au. 2025 May 19;5(6):2593–605. doi: 10.1021/jacsau.5c00210 (PMC12188485; doi:10.1021/jacsau.5c00210)
Supplement: Supplementary file 2 [file au5c00210_si_002.pdf]

[redacted]

[redacted] **INTRODUCTORY CHEMISTRY LABORATORY, FALL 2018**

1. General Information

Laboratory Times: [redacted]

Laboratory Locations: [redacted]

Attendance is mandatory at all laboratory meetings. See the schedules and section assignments on [redacted]. Room assignments may change throughout the semester as construction work requires.

Credit Hours: 1

[redacted]

[redacted]

Teaching Assistants: See [redacted] for TAs and their office hours. TA office hours are held in [redacted]

A Note about correspondance: Due to the large number of students enrolled in the course (over 1700 students in about 74 sections), we suggest you take any questions or concerns to your section TA first. If an answer or solution cannot be found, then contact a head TA. If an answer or solution is still elusive, then contact [redacted].

2. Course Description

Start your exploration of chemistry here! This course is an introduction to experimental chemistry, developing laboratory skills and safety. Students plan and implement chemistry experiments in

cooperative 4-person teams using a guided inquiry approach. Process skills include developing procedures, data analysis, oral and written communication. Mathematica is used as a computational tool.

3. Course Materials

Notebook "Introductory Chemistry Laboratory" notebook for [redacted] Used lab notebooks are not allowed. No other notebooks allowed.

Matematica Licenses are available at the Customer Service desk of the bookstore.

Safety Goggles 3M tech safety goggles are available at the Bookstore and local retailers as "3M Clear Plastic Chemical Impact Goggle". No other models allowed.

Lab coat Available at the bookstore

Website: [redacted]

Other: Please bring pen, calculator (with log and ln functions; graphing not necessary), and your laptop to every lab. meeting.

Recommended Reading These titles are not required, but may help you develop your scientific writing skills. If you plan on majoring in Chemistry, you may wish to invest in your own copies.

(1) *The ACS Style Guide: Effective Communication of Scientific Information.* A.M. Coghill and L.R. Garson, Eds.; American Chemical Society: Washington, D.C., 2006. https://pubs.acs.org/isbn/9780841239999

(2) J.A. Penchenik, H.B. Davis, and J.F. Tyson. *A short guide to writing about chemistry.* Pearson Education: Canada, 2009.

4. Student Learning Outcomes

- Appropriately choose and utilize various laboratory techniques and equipment
- Keep a laboratory notebook using standard notebook format and etiquette
- Write simple programming code in Mathematica and understand the value and importance of programming in chemistry
- Develop and implement experimental methods
- Gather, analyze and evaluate data
- Understand underlying chemical principles and apply these principles to experimental data
- Work collaboratively with peers to develop ideas and critically analyze conclusions
- Effectively communicate experimental ideas and chemical principles orally and in writing

5. Grading

There will be four projects throughout the semester that encompass a variety of different activities and assignments to help you meet the course objectives. TAs assess all assignments against a standardized rubric. General rubrics for plans, summaries, presentations, student evaluations and lab report write-ups will be available on [redacted]. Your final grade is determined as the total number of points you have earned divided by total possible points. There will be no curve in this course.

Prelab assignments: 80 points (1 prelab worth 5 points, 3 prelabs worth 25 points each)

For each project, you will complete an individual pre-lab assignment to help prepare you for working with your team to develop an experimental plan. The prelab assignments will be completed in Mathematica to learn and practice coding in Mathematica. Can be submitted late for a penalty.

Experimental Plans and Summaries: 64 points (5 plans worth 5 points each, 2 summaries worth 6 points each, 3 summaries worth 9 points each)

For each experimental day, you will work in a team to plan out your experiment and summarize each experiment. The purpose of planning and summarizing experimentation is to get you thinking systematically about how you will approach solving a scientific question. Working on plans and summaries during class time will provide you opportunities to engage in team work, a valuable skill for virtually any career. Plans and summaries will be completed as a group and turned in during class time, so they cannot be made up.

Postlab assignments: 200 points (4 postlabs worth 50 points each)

Post-lab assignments will be one way you will communicate your projects' findings and will help you make connections between your experimental approach and chemical principles and/or Mathematica programming. All post-lab assignments will be turned in individually. Can be submitted late for a penalty.

Quizzes: 60 points (3 quizzes worth 20 points each)

At the end of each project you will take a quiz to assess your understanding of chemical principles and laboratory techniques related to the project (Projects 1 and 2 will be combined). These multiple choice quizzes will be taken at the beginning of the class period and completed individually. These quizzes cannot be made up.

Presentations: 50 points (2 presentations worth 25 points each)

Each team will present the results of their experimentation to their TA and lab section. The purpose of these presentations is to practice your public speaking and hone your ability to explain chemical phenomena to your peers. Following the presentations, your TA will hold a whole-class discussion about each team's approach to the project and help you make connections between your project, chemistry, and other real-world applications. Presentations cannot be made up.

Peer Assessments: 48 points (3 evaluations worth 16 points each)

You will be working in a team every day in lab, and you will have the opportunity to provide written feedback and communicate to your team members on your perceptions of the team dynamics project. Your TA will also provide team members feedback on their efforts in the group. The purpose of providing feedback to students on their teamwork is to help students learn and improve on their skills in collaborative work. Each student will receive a peer assessment score for each project (projects 1 and 2 will be combined). Peer assessments will be completed during lab.

6. Succeeding in This Course

The purpose of the general chemistry laboratory is to explore chemical concepts, to begin to develop your chemical intuition, to give you opportunities to practice scientific communication, and to help you develop practical skills that are useful for scientists in general (e.g. designing experiments to answer questions) or chemists in particular (e.g. performing titrations).

You are expected to take responsibility for your own learning and be an active participant in all aspects of lab. This can be overwhelming, but you can all rise to the challenge! Below are some of the ways that students can prepare for and be successful in this course.

- Read suggested articles and do research related to the planning questions before coming in to lab
- Be an active participant in lab during planning, experimenting, and presenting
- Wear appropriate attire during lab: long pants, socks, close toed shoes, lab coat, goggles
- Prepare to do about 3 hours of work outside of lab each week on average. Some weeks will require more out-of-class work, and some will require less.
- Find time to meet with your group outside of lab to work on presentations
- Work on assignments ahead of the due date so you can attend office hours and get help when needed.
- Don't get in your own way! Pride, shyness, low self-esteem, and other traits can prevent students from asking questions or speaking up. A common consequence of this silence is not learning as much as you could have. Your instructor and TAs are here to help, but we can't do that if we don't know what you are confused about.

It is very difficult to succeed if you do not understand the expectations of the course. If you are confused about what you need to do, just ask! Questions about our expectations of what you should be doing before, during, and after lab are always welcome.

7. Safety

Safety is the most important goal of every laboratory experiment! Never complete an experimental step if you do not know what you are doing, why you are doing it, and how to minimize any risks involved. When in doubt, ask!

Safety rules must be observed in this laboratory course at all times. Any of the laboratory staff can immediately dismiss a student who poses a danger to him(her)self or to anyone else. This includes proper attire. A student who is unwilling to follow directions from an instructor or TA also poses a safety hazard, and may be dismissed.

Remember that all injuries must be reported immediately to a TA or lab supervisor, and that cleanliness/tidiness and proper disposal of leftover chemicals are part of good safety practices.

8. Attendance and Make-up Opportunities

Since you will be working in collaborative teams during lab, it is also essential you show up to lab on time and do not miss any lab sessions. There is no make-up week for missed experimental work. Should you miss an experiment for an excused reason, you will still have to complete the

required assignments (prelabs/postlabs/etc.) independent of your group and may have alternative assignments depending upon the day missed. There are certain assignments that cannot be made up, even for an excused absence. The following constitute potentially excused absences: family emergency, death in the family, severe individual illness, and participation on a [redacted] varsity athletic team that requires you to travel during your lab meeting. Your TA and lab group members must be notified by e-mail of the absence prior to lab unless it is due to a completely unforeseeable reason, in which case, you will have 48 hours after the missed lab to notify the instructor or TA. If you missed lab for any unexcused reason you may receive a 0 or a grade penalty, depending upon the assignment. Since there is no opportunity to make-up experimental work, and since laboratory courses are inherently experiential in nature, if you miss more than 2 class meetings (both experiments and workshops count as meetings), even with excused absences, you may be asked to withdraw from the course. If the withdraw date has passed, you will receive a failing grade.

9. Re-grades

If at any time you feel that any of your work was graded incorrectly, please don't hesitate to bring it to our attention by using the following protocol:

(1) On a separate piece of paper, write a short note (politely, and with complete sentences!) explaining what writing or work you would like to be reassessed, and the reason why you think the original grade was incorrectly assigned.

(2) Attach the note to the original assignment and give both to your TA

(3) You will be contacted by e-mail within one week with the regrade decision. You may also be e-mailed if it is necessary to clarify something in your note.

Regrades will never be decided while the student involved is physically present. That creates unfair pressure on TAs or instructors to make a quick decision. Also remember to not make any changes to the assignment itself before resubmission. Any change will be considered a violation of academic integrity and will be penalized accordingly. **Any time you ask for a re-grade on completed work, we reserve the right to regrade any portion of that assignment, not just the part requested.**

Requests for regrades must be addressed within two weeks of receiving your score on any assignment; no requests for regrades will be humored after that deadline. Because of the need to submit final grades to the registrar quickly at the end of the semester, no regrade requests are possible for the last postlab of the semester.

10. Honor Code

Because we want all students to learn to be more like scientists, whose reputations depend upon the trustworthiness of their work, the Honor System is taken very seriously in this course. By writing the pledge on your work, you are stating: [redacted] Do not hesitate to ask the TAs or your instructor for assistance throughout the semester.

In this course, academic dishonesty includes "fudging" laboratory data such as weights, melting points, etc. The fabrication of scientific data and information is an egregious violation of the

underpinnings of scientific inquiry and the scientific method. A violation of this policy will be treated as outlined below.

Penalties for Academic Integrity Violations

A first or second instance of academic dishonesty (cheating or plagiarism) will result in a grade of 0% on the experiment/assignment during which the dishonesty took place. A third instance of academic dishonesty will result in automatic failure of the course without option to withdraw. Instances of dishonesty may be reported to the [redacted].

When it comes to plagiarism, it does not matter if you plagiarized or if you knowingly allowed someone to plagiarize off of you, the penalty is the same. When it comes to cheating, it does not matter if you cheated or if you knowingly allowed someone to cheat off of you, the penalty is the same.

11. Extra Credit

There is none. Please do not ask. Focus on doing quality work on remaining assignments.

12. Academic Concerns

Students are encouraged to discuss concerns about academic issues with faculty members according to the following sequence. If the concern is about a grade, be advised that it is your responsibility to keep all graded papers in the event that there is a discrepancy at the end of the semester.

(1) A student should informally discuss any concerns with the instructor or TA involved. In most cases, this discussion should result in the clarification of any misunderstandings and/or a satisfactory resolution for all parties.

(2) If a student is not satisfied with the outcome of this informal process, he or she should formally present his/her concerns in writing to an instructor (e-mail preferred).

(3) A meeting between the student and instructor will be organized to discuss these concerns.

(4) Following this meeting, the instructor will carefully consider the situation and present her decision in writing to the student.

(5) If a student is not satisfied with the outcome of this formal process, he/she should consult with the Department Chair.

Students who skip steps in this sequence will probably not be satisfied with the outcome of their action.

13. Students with Disabilities

All students with special needs requiring accommodations should present the appropriate paperwork from the [redacted]. It is the student's responsibility to present this paperwork in a timely fashion and follow up with the instructor about the accommodations being offered. Accommodations for quiz-taking(e.g., extended time) should be arranged at least 3 days before a quiz.
